# Imaging neuroinflammation in individuals with substance use disorders

**DOI:** 10.1172/JCI172884

**Published:** 2024-06-03

**Authors:** Xinyi Li, Astrid P. Ramos-Rolón, Gabriel Kass, Lais S. Pereira-Rufino, Naomi Shifman, Zhenhao Shi, Nora D. Volkow, Corinde E. Wiers

**Affiliations:** 1 Center for Studies of Addiction, University of Pennsylvania Perelman School of Medicine, Department of Psychiatry, Philadelphia, Pennsylvania, USA.; 2 Departamento de Morfologia e Genética, Escola Paulista de Medicina, Universidade Federal de São Paulo, São Paulo, Brazil.; 3 Laboratory of Neuroimaging, National Institute on Alcohol Abuse and Alcoholism, NIH, Bethesda, Maryland, USA.

## Abstract

Increasing evidence suggests a role of neuroinflammation in substance use disorders (SUDs). This Review presents findings from neuroimaging studies assessing brain markers of inflammation in vivo in individuals with SUDs. Most studies investigated the translocator protein 18 kDa (TSPO) using PET; neuroimmune markers myo-inositol, choline-containing compounds, and N-acetyl aspartate using magnetic resonance spectroscopy; and fractional anisotropy using MRI. Study findings have contributed to a greater understanding of neuroimmune function in the pathophysiology of SUDs, including its temporal dynamics (i.e., acute versus chronic substance use) and new targets for SUD treatment.

## Introduction

Substance use disorders (SUD) cause substantial economic and public health challenges globally. In the United States, over 46.3 million Americans aged 12 years or older were affected by SUDs in 2021. SUDs are chronic relapsing multifactorial disorders characterized by a range of structural and functional changes in the brain, as revealed by preclinical and human neuroimaging studies (1). The heuristic framework for SUD outlines a three-stage recurring cycle of binge/intoxication, withdrawal/negative affect, and preoccupation/anticipation that is governed by three functional domains (incentive salience, negative emotional states, and executive functions) (2). Engagement of the mesocorticolimbic system, consisting of the ventral tegmental area (VTA), striatum, prefrontal cortex, amygdala, and hippocampus, in the binge/intoxication stage heightens the incentive saliency of misused drugs, forms conditioned responses to drug-related cues, and facilitates maladaptive habit formation/compulsive drug behaviors (2). Discontinuing substance use produces symptoms of withdrawal and negative effects, which are mediated by brain regions involved in stress response and emotional regulation (i.e., the amygdala) (2). Reconciling memories of previous drug use (amygdala and hippocampus), craving (prefrontal cortex and striatum), and decreasing executive control (prefrontal cortex) in the preoccupation/anticipation stage subsequently leads to relapse and perpetuates SUD (3). These functional and structural neuroadaptations may be attributable to neuroinflammation following prolonged substance use that increases neurotoxicity and promotes neurodegeneration in individuals with SUD (4).

Inflammation is a biological process triggered by a variety of harmful insults, such as infection, ischemia, stress, and trauma (5, 6). In the CNS, a key element of neuroinflammation is the activation of immune-specific cells (i.e., microglia and astrocytes) that synthesize inflammatory mediators and promote leukocyte recruitment. Activation of the TLRs on microglia by pathogen-associated or internal damage-associated molecular patterns initiates the immune response. The activated TLRs promote the recruitment of NF-κB, resulting in the production of different mediators, such as proinflammatory cytokines (e.g., IL-6, IL-1, and TNF-α), type I interferon (IFN-β), chemokines (CCL5), and cyclooxygenase-2 (COX-2) (7, 8).

Studies on postmortem markers of inflammation revealed increased markers of microglia in the cingulate cortex, VTA, amygdala, and midbrain of individuals with alcohol use disorder (AUD) compared with those of nondependent controls (9, 10). They also showed higher expression of TLRs and downstream signaling cascade NF-κB in the orbital frontal cortex and higher concentration of proinflammatory cytokines (i.e., monocyte chemoattractant protein-1) in the VTA, hippocampus, and amygdala (10–12). Similarly, chronic opioid exposure has been associated with upregulation of glial activation and immune response pathways (13). While the initial stage of neuroinflammation may be beneficial and protective, overactivation of TLRs in prolonged inflammation promotes cytotoxic changes, including the development of brain edema, gliosis, blood-brain barrier disruption, astrocyte proliferation, oxidative stress, and changes in cell survival transcription factors (14). Neuroinflammation was associated with neurodegeneration in neuropsychiatric disorders (7, 15) and may mediate structural and functional deficits that contribute to SUD.

**Figure 1:**
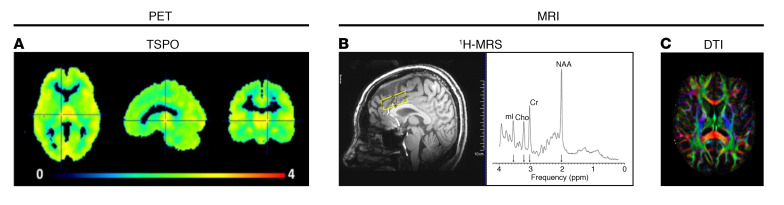
Imaging markers of neuroinflammation. (**A**) PET image of TSPO radiotracer [^11^C]PBR28 (image reproduced with permission from Kim et al., 2018, ref. 21). (**B**) Representative image of ^1^H-MRS voxel placement in the dorsal anterior cingulate cortex and ^1^H-MRS spectra of inflammation markers, NAA, cho, and mI (spectra image reproduced with permission from Blüml, 2013, ref. 130). (**C**) DTI fractional anisotropy (FA) color map. Cho, choline-containing compounds; Cr, creatine; DTI, diffusion tensor imaging; ^1^H-MRS, magnetic resonance spectroscopy; mI, myo-inositol; NAA, N-acetyl-aspartate; TSPO, translocator protein 18 kDa.

The present Review focuses on the link between SUD and neuroinflammation based on human neuroimaging studies using PET and MRI (Figure 1). We will discuss findings that pertain to common substances of misuse, including alcohol, nicotine, opioids, cannabis, and stimulants.

## PET imaging: TSPO

Studies using PET to examine neuroinflammation have mainly focused on measures of translocator protein 18 kDa (TSPO) expression and binding. TSPO is a transmembrane protein localized in the outer mitochondrial membrane that mediates essential mitochondrial functions, such as regulating cholesterol transport steroid hormone synthesis, apoptosis, and cell proliferation (16). Within the CNS, TSPO is primarily expressed in microglia and reactive astrocytes, which are immune cells integral to the brain, and serves as a marker for immune system activation (4, 17). Several PET radiotracers have been developed to detect TSPO, including [^11^C]PK11195, [^11^C]PBR28, [^11^C]DAA1106, and [^18^F]FEPPA (4, 17). A summary of studies measuring TSPO in participants with SUD compared with nondependent controls is provided in Table 1.

### Alcohol.

Thus far, three studies have compared TSPO binding in AUD, and all found, contrary to their hypothesis, lower [^11^C]PBR28 volumes of distribution (V_T_) in individuals with AUD compared with that in healthy, nondependent control groups (meta-analyzed in ref. 18). Kalk et al. were the first to report lower hippocampal [^11^C]PBR28 V_T_ in individuals with AUD compared with individuals acting as controls and nonsignificant trends for lower [^11^C]PBR28 V_T_ in the midbrain, thalamus, cerebellum, and anterior cingulate cortex (ACC) (19). However, the recruited individuals with AUD were more likely to be smokers (8 of 9) than the people in the control group (5 of 20), and differences in smoking status may had confounding effects on the study results. The findings are concordant with those of another study that reported lower [^11^C]PBR28 V_T_ in individuals with AUD than in controls matched for smoking status. Post hoc analyses examining regional differences in [^11^C]PBR28 V_T_ revealed a significant effect of AUD in the cerebellum and trends for the frontal cortex, striatum, and hippocampus. Additional exploratory analysis indicated that [^11^C]PBR28 V_T_ negatively correlated with alcohol dependence severity and number of drinks per day in the past month (20). Kim et al. (21) found no significant group differences in whole-brain [^11^C]PBR28 binding between individuals with AUD and controls, but when separated by TSPO genotype (medium vs. high-affinity binding), those with the medium-affinity genotype and AUD had lower [^11^C]PBR28 V_T_ than controls in the whole brain, gray matter, white matter, hippocampus, and thalamus. Although the study did not match for smoking status (10 of 19 smokers in the AUD group and no smokers in the control group), additional analyses showed no effects of smoking status on TSPO binding within the AUD group.

Despite the directionally consistent findings, they should be interpreted with the following considerations. Laurell et al. (22) reanalyzed data collected by Hillmer et al. (20) and separated total V_T_ into ligand-specific distribution volume (V_S_) and non-displaceable-binding distribution volume (V_ND_). AUD compared with healthy controls demonstrated significantly lower V_ND_ but no differences in V_S_ (22), raising the possibility that differences in [^11^C]PBR28 V_T_ between patients and controls may be attributable to non-displaceable- instead of ligand-specific binding. Second, participants’ blood cholesterol and triglyceride levels correlated inversely with [^11^C]PBR28 (21, 23), as cholesterol binds to TSPO for transport during steroid synthesis (24). Dyslipidemia is evidenced in AUD (25), and the lower [^11^C]PBR28 binding reported by PET studies may reflect greater competition from cholesterol for binding to TSPO in AUD. Third, rs6971 TSPO genotype (high-affinity binders, low-affinity binders, and mixed-affinity binders) has been shown to alter the affinity of [^11^C]PBR28 for TSPO (22) and lipid levels (25) and may have implications for influencing the relationship between TSPO and AUD status (21). Thus, more work is needed to conclusively identify the mechanisms underlying lower TSPO in AUD.

A study that evaluated the effects of an acute oral alcohol challenge (adjusted to achieve a blood alcohol level of 80 mg/dL) in healthy volunteers found that alcohol increased [^11^C]PBR28 V_T_ by an average of 12% (26). Alcohol-induced increases in [^11^C]PBR28 V_T_ correlated negatively with the subjective effects of alcohol (26). This is in line with findings from a previous study in baboons that also found higher [^18^F]DPA-714 V_T_ (58%–138%) in animals exposed to an acute intravenous alcohol infusion of 0.7–1 g/L compared with alcohol-naive animals (27). Although TSPO levels were reduced 7–12 months after the alcohol infusion in the alcohol-exposed animals, levels remained higher than those in alcohol-naive animals. The mechanisms underlying the differential effects of acute versus chronic alcohol administration on brain TSPO levels require further elucidation. It has been postulated that chronic microglial activation in response to chronic alcohol use diminishes TSPO levels and the subsequent reduced immune response in AUD contributes to an enhanced susceptibility to diseases (20). In line with this, individuals with AUD showed lower peripheral cytokine response to stimulation with LPS than controls (20).

### Tobacco.

One study in nicotine users showed 16.8% lower whole-brain [^11^C]DAA1106 binding (measured as standard uptake values [SUVs]) in smokers during smoking satiety (i.e., having smoked ~15 minutes prior to scanning procedures) compared with nonsmokers on all volumes of interest (VOIs): amygdala, caudate, accumbens, hippocampus, putamen, and thalamus (28). A subsequent study showed that [^11^C]DAA1106 SUVs in smokers remained low, even following overnight (~12 hours) abstinence in the same VOIs as the previous study (29). Higher levels of cigarette exposure, as indicated by the depth of inhalation (29) or cigarettes per day (28), were associated with lower [^11^C]DAA1106 binding, which was interpreted as reduced TPSO levels. The type of cigarette also altered TSPO levels, as three-way comparisons showed that SUV was highest in nonsmokers, in the middle in nonmenthol cigarette smokers, and lowest in menthol cigarette smokers (28, 29). In contrast, another study found no significant differences in [^11^C]PBR28 binding (measured as V_T_) between smokers (abstinent for 2–14 hours before the scan) and nonsmokers in whole brain or any of the VOIs (30). The inconsistent study findings may be attributable to differences in radioligands ([^11^C]PBR28 vs. [^11^C]DAA1106) or quantification methods (SUV vs. V_T_). V_T_ is the gold-standard quantification method that, unlike SUV, accounts for plasma radioligand concentration and potential differences in radioligand delivery to the brain (31). Yoder et al., retrospectively compared SUVs and V_T_ from [^11^C]PBR28 PET scans acquired in baboons at baseline and at varying time points following LPS injections. Although regional SUV and V_T_ were highly correlated, the slope of their relationships varied across individuals and ROIs, suggesting discrepancies between SUV and V_T_ (31).

### Stimulants.

An early study using the TSPO tracer [^11^C]*(R)-*PK11195 demonstrated higher binding (measured as binding potential) in individuals with a history of methamphetamine use disorder (MUD; abstinent 0.5–4 years) than healthy volunteers in the midbrain, striatum, thalamus, orbitofrontal cortex, and insular cortex (32). *(R)-*PK11195 binding potential was negatively correlated with the duration of abstinence in the midbrain, striatum, and thalamus, suggesting that dysregulation in neuronal immune response may normalize with prolonged abstinence (32). A later study by London et al., quantifying TSPO binding with SUV and newer generation of TSPO PET radiotracers, [^11^C]DAA1106, found no differences between individuals with MUD in early abstinence (<6 months) and healthy controls in whole-brain TSPO levels or any of the examined VOIs (33). Similarly, Rathitharan et al. (34) measured [18C]FEPPA V_T_ and did not demonstrate significant group differences in TSPO binding. The inconsistent findings may be attributable to the high nonspecific binding of [^11^C] *(R)-*PK11195 compared with the newer TSPO PET tracers, PET quantification methods, or study differences in participant characteristics (e.g., early vs. prolonged abstinence or MUD severity) and additional studies are warranted.

A study of cocaine use disorder (CUD) found no significant differences in [^11^C]PBR28 V_T_ between recently abstinent cocaine-dependent individuals and nondependent controls in the midbrain, striatum, cerebral cortex, ACC, medial temporal lobe, or cerebellum (35).

### Cannabis.

In the only clinical study examining the effects of cannabis on TSPO, long-term cannabis users (use >4 times/week for the past 12 months) had higher brain [^18^F]FEPPA V_T_ than controls in total and across the dorsolateral prefrontal cortex (dlPFC), medial prefrontal cortex, temporal cortex, ACC, cerebellum, and gray matter as a whole. More prominent effects were observed in a subset of individuals who met the diagnostic criteria for cannabis use disorder. Exploratory analysis in cannabis users demonstrated that [18F]FEPPA V_T_ negatively correlated with lifetime cannabis use, which remained trending but no longer significant after controlling for sex, but not cannabis craving and dependence severity (36).

### Opioids.

To the best of our knowledge, no human studies have been published on the effect of opioids on brain TSPO binding.

## MRI: magnetic resonance spectroscopy

Proton magnetic resonance spectroscopy (^1^H-MRS) is a noninvasive neuroimaging technique that uses the MRI scanner to measure local concentrations of neuroinflammatory biomarkers: (a) myo-inositol (mI), an organic molecule present in glial cells that serves as a marker for glial cell activation and neuroinflammation; (b) N-acetyl aspartate (NAA), a neuronal marker, decreasing levels of which are indicative of neuronal dysfunction; (c) choline-containing compounds (Cho), which reflect membrane turnover; and (d) glutathione (GSH), which is involved in the cellular defense against oxidative stress and provides information on cellular inflammatory processes. Levels of brain metabolites are commonly expressed relative to creatine (Cre) or water, but absolute measures are also reported (37, 38). A summary of ^1^H-MRS studies that included these inflammatory markers in individuals with SUDs compared with nondependent controls is provided in Tables 2 and 3.

### Alcohol.

A meta-analysis of 43 ^1^H-MRS studies in individuals with AUD showed lower levels of NAA and Cho but no differences in mI compared with nondependent healthy volunteers (39). Specifically, lower levels of NAA and Cho were recorded in the frontal cortex, cerebellum, hippocampus, and frontal and parietal white matter. Lower Cho was also observed in the temporal cortex and thalamus, and NAA in the ACC (39). The concentrations of brain metabolites in individuals with AUD depend upon several factors, including individual drinking habits and duration of abstinence. For example, in individuals with AUD, the number of heavy drinking days in the 14 days prior to the MRI scan was inversely associated with dorsal ACC NAA/water (40), and more recent drinking correlated with lower NAA and Cho levels in frontal and thalamic regions (41). Lower hippocampal NAA (42) and greater thalamic mI levels (43) were observed in recently detoxified individuals with AUD though these measures recovered with abstinence.

Acute alcohol administration (0.65 g/kg in female users and 0.75 g/kg in male users) in healthy volunteers decreased Cho and mI in the frontal cortex and cerebellum 1.5 hours after ingestion compared with baseline but rebounded by 12 hours (44). Another study in which alcohol was infused intravenously to a target breath alcohol concentration of 60 mg/dL showed a similar reduction in Cho in the occipital cortex within an hour of administration but, in contrast to the previous study, found a trend for increasing mI as well as a decrease in NAA (45). Measurement of brain metabolites in the descending limb of the blood alcohol curve, approximately 4–5 hours following alcohol administration, demonstrated significant elevation in Cho/Cre and glutathione/Cre (GSH/Cre) in the thalamus but no effects on mI/Cre or NAA/Cre (46). The inconsistent findings of these studies raises questions about the temporal (i.e., ascending vs. descending limb) and regional (i.e., differing brain regions) effects of alcohol on brain metabolites.

### Tobacco.

Findings in individuals who smoke tobacco showed lower levels or no group differences in NAA, Cho, and mI levels compared with healthy controls (47). Low NAA levels, in particular, have been associated with poorer decision-making and higher impulsivity in smokers (47). Studies examining the role of abstinence and smoking severity on neuroinflammatory markers have yielded mixed results. Some reported no differences in neural metabolites between nicotine-deprived (24–72 h) and satiated states (48–50) or between individuals who smoke daily (>5 cigarettes/day) and those who smoke intermittently (1–4 cigarettes on at least 1 day per week) (51). Another study showed a positive correlation between lifetime tobacco exposure (pack-years) and ACC Cho levels (52).

Cigarette smoking often occurs concomitantly with heavy alcohol consumption and may exacerbate impairments in neural functioning in individuals with AUD. In comparison with nonsmoking individuals with AUD, those who smoked tobacco demonstrated lower NAA and Cho levels (53). Brain metabolite levels normalized with abstinence from alcohol in nonsmoking individuals with AUD but remained significantly reduced at 1 month in those who smoked (54). Abstinence-associated changes in Cho and mI levels correlated with improvements in visuospatial memory (54). Therefore, reductions in brain metabolites in smoking individuals with AUD may adversely affect their neural recovery (54).

### Stimulants.

Studies in individuals with MUD generally demonstrated lower NAA levels in frontal regions, including the ACC, but results for Cho and mI have been inconclusive (Table 3). Low NAA/Cre and Cho/Cre levels in the dlPFC and ACC correlated with neurocognitive impairments in individuals with MUD (55, 56). Greater duration of methamphetamine use was associated with greater reductions in NAA and mI (57). Prolonged abstinence may normalize brain metabolite levels. Thus, short-term abstinence from methamphetamine (1–6 months) resulted in lower NAA/Cre and higher mI/Cre compared with controls, but no differences were observed during longer-term abstinence (1–5 years) (58). Furthermore, the duration of abstinence was positively correlated with ACC NAA/Cre levels (58). Recovery may be slow, as one study demonstrated a persistent reduction in dlPFC NAA/Cre in individuals following approximately 5 weeks of abstinence (57).

An acute intravenous dose of cocaine (0.2 and 0.4 mg/kg) in occasional cocaine users increased NAA and Cho levels as compared with intravenous placebo (59). However, a comparison of individuals with CUD and healthy controls yielded no differences in ^1^H-MRS makers of neuroinflammation (Table 3).

### Cannabis.

Administration of cannabis (300 μg/kg of Δ-9-tetrahydrocannabinol [THC]) versus placebo to occasional and heavy cannabis users increased mI/Cre in the striatum and ACC and NAA/Cre in the ACC, but only in the occasional users and not in the heavy users, suggesting cannabis tolerance in the latter group (60). Studies comparing cannabis users and controls generally indicated a decrease in mI (except for Muetzel et al., ref. 61, who found higher mI in female users compared with female nonusers but no differences between male users and nonusers) and no group differences in NAA and Cho levels (Table 3). Low mI levels significantly correlated with higher problematic drug use behavior and cannabis dependence (62), but no significant correlations were observed between brain metabolite levels and marijuana exposure (i.e., age of onset and total lifetime use) (63, 64). Cannabis-associated changes in brain metabolites may adversely affect neuropsychological performance. As such, thalamic mI/Cre levels were associated with greater cognitive impulsivity (64).

### Opioids.

Most ^1^H-MRS studies in individuals with opioid use disorder (OUD) demonstrated low brain levels of NAA (Table 3). Methadone-treated individuals with OUD showed lower mI in the ACC than those maintained on buprenorphine, and the dose of methadone correlated negatively with mI and positively with NAA (65).

## MRI: diffusion tensor imaging

Diffusion-tension imaging (DTI) is an MRI technique that measures the motion and diffusion of water molecules within tissues and assesses white matter microstructures. Fractional anisotropy (FA) is calculated from DTI and indexes the nonuniformity of water diffusion. High FA reflects selective diffusion along specific directions due to the presence of impermeable or semipermeable walls in the white matter (66). Although FA is not a direct marker of neuroinflammation, low FA may be indicative of demyelination, axonal loss, and blood-brain barrier permeability in neuroinflammatory conditions (66, 67).

### Alcohol.

An analysis of 25,378 participants from the UK Biobank reported a negative correlation between weekly alcohol intake and FA in the corpus callosum and fornix (68, 69). Furthermore, case-control studies consistently show lower FA in individuals with AUD compared with healthy controls, suggestive of aberrations in brain white matter microstructure and neuroinflammation with chronic alcohol consumption (Table 3). During early abstinence (2–3 weeks) FA was further diminished (70), but longer-term abstinence tended to increase FA, especially in the right cingulum and hippocampus (71, 72). In contrast, Fortier et al. (73) found widespread reductions in FA, including in frontal, temporal, parietal, and cerebellar white matter in individuals with AUD with an average of 25 years of misuse compared with controls following 5 years of alcohol abstinence (Table 4).

Low FA may have neuropsychological consequences that impact AUD treatment outcomes. Monnig et al. (74) associated lower FA with greater conditioned brain response to alcohol cues in the frontoparietal and corticothalamic networks, as measured by functional MRI. Lower FA in the corpus callosum and corona radiata has also been associated with greater impulsivity and poorer decision-making in individuals with AUD (75, 76). Finally, FA measurements have been shown to predict relapse in individuals with AUD: those who relapsed 1 (77) or 6 months (78) after treatment had lower baseline FA in the corpus callosum (77), frontal white matter tract (78), and stria terminalis (77) than those who successfully abstained from alcohol.

### Tobacco.

Studies comparing FA between smokers and nonsmokers have shown inconsistent results (Table 5). One study reported increased FA in the prefrontal white matter, cingulum, and corpus callosum (79), while another found lower FA in the cingulum of smokers compared with nonsmokers (80). Interestingly, both studies found a negative association between FA and tobacco exposure in these distinct regions (79, 80). One possible explanation that reconciles these inconsistencies is that tobacco smoking increases FA in early adulthood, which then declines with continual smoking in later life (81).

### Stimulants.

A meta-analysis in individuals with stimulant use disorder (i.e., CUD and MUD) showed lower FA compared with controls in the corpus callosum, particularly the genu, and the frontal white matter, all with small-to-moderate effects (82). Studies examining FA in individuals with MUD are listed in Table 5 and generally reported either lower FA compared with controls or no group differences. Low FA was found to contribute to impaired performance on neurocognitive tasks (e.g., performance on the Stroop Attention Test or Wisconsin Card Sorting Test) in individuals with MUD (83, 84).

A meta-analysis showed lower FA in CUD than control individuals in the corpus callosum with small-to-moderate effect size, and in the anterior thalamic projections and striatum at the level of a trend (85). Several studies associated lower FA with higher impulsivity (86, 87) and altered reward signaling (88), which have been thought to contribute to poorer CUD treatment outcomes. In treatment-seeking individuals with CUD, higher baseline FA prior to treatment initiation was positively associated with a longer duration of abstinence (89). He et al. (90) found low FA in the frontal cortical tracts (e.g., corpus callosum, superior longitudinal fasciculus, and inferior frontal-occipital fasciculus) and the frontal white matter in individuals with current but not past CUD, suggesting that abstinence may restore FA levels.

### Opioids.

Multiple studies found lower FA in individuals with OUD compared with healthy controls (Table 5), except for Sun et al. (91) who reported higher FA. Specifically, a meta-analysis of extant literature reported low FA in the frontal subgyral area, including the cingulum and superior longitudinal fasciculus in individuals with OUD compared with controls (92). Exposure to methadone contributed to, but did not fully account for low FA (93), as individuals with OUD who were not maintained on medications for treating OUD (methadone and buprenorphine) also showed low FA (94). In a comparison of individuals with fewer than 10 years or 10–20 years of heroin use, a longer duration of opioid exposure was associated with more widespread reductions in FA, particularly in the corpus callosum, thalamic radiation, and parietal, frontal, and temporal tracts (95). Cessation of opioid use partially restored FA measures (96, 97).

Several studies associated lower FA with impaired decision-making and impulsivity on the Iowa Gambling Task (95, 98). Furthermore, another study reported a negative relationship between FA in the frontostriatal tract and opioid craving in individuals with OUD (96). The role of FA in these neuropsychological domains could have implications for treatment outcomes. In individuals undergoing methadone treatment, those who relapsed after 6 months demonstrated lower FA in the internal and external capsules and the corona radiata than those who were abstinent (99).

## MRI: linking peripheral inflammatory markers and brain function

Chronic inflammation contributes to neurodegeneration in normal aging and neurodegenerative diseases, including Alzheimer’s and Parkinson’s disease (100–102). Peripheral inflammatory markers (i.e., white blood cells, high-sensitivity C-reactive protein, and fibrinogen) were found to associate with brain signatures of aging, including reduced total brain and gray matter volume (103). Similarly, several studies, as summarized below, have shown that inflammatory markers mediate the neurobiological consequences of substance misuse.

### Alcohol.

Increased intestinal permeability due to alcohol consumption may lead to the leakage of proinflammatory LPS into the systemic circulation, triggering an inflammatory cascade. In comparison with healthy controls, individuals with AUD showed intestinal hyperpermeability, higher plasma LPS concentrations, and higher levels of the proinflammatory cytokines TNF-α and IL-8 (104), which correlated positively with alcohol craving, suggesting a gut-brain interaction in individuals with AUD (104). TLR4 is a pattern recognition receptor that identifies pathogens and triggers inflammatory responses. Methylation of the *TLR4* gene moderated the relationship between alcohol use severity and brain gray matter volume, such that greater alcohol severity was associated with lower precuneus and inferior parietal gray volumes in individuals with low (but not high) methylation (105).

### Stimulants.

Higher levels of IL-6 but not IL-1β or IL-10 were found in individuals with MUD compared with healthy controls (106), which, in individuals with MUD, was associated with disruptions in striatal-limbic and cortico-striatal resting-state functional connectivity (106).

### Opioids.

In individuals with OUD, 12 weeks of methadone treatment significantly decreased plasma cytokine levels and improved behavioral performance on a memory task (107). These changes in inflammatory markers and memory capacity were strongly correlated: the concentration of TNF-α correlated negatively with visual memory capacity, and concentration of IL-6 correlated negatively with verbal memory and decayed recall indices (107). A follow-up study also associated higher IL-6 levels during methadone treatment with poorer medication compliance and drop-out (108), reflecting adverse effects of neuroinflammation on real-life behaviors. In concert, findings from these studies highlight the role of inflammation in neural functioning and its potential implications for OUD treatment outcomes.

## Discussion and future directions

Here, we reviewed the human literature on PET and MRI markers of neuroinflammation in SUDs. PET studies with tracers that bind to TSPO, a marker of microglial activation, demonstrated consistent upregulation in response to neuroimmune challenges (109) and to acute alcohol administration (26). However, differential effects have been reported in SUDs. Chronic alcohol exposure in individuals with AUD is consistently associated with lower TSPO than in nondependent controls (meta-analyzed in ref. 18), suggesting temporal dynamics of acute inflammatory insults versus downregulation of TSPO in response to chronic alcohol exposure. Tobacco smoking was also associated with low brain TSPO levels in two of three studies, whereas cocaine, methamphetamine, and cannabis users were found either to have higher brain TSPO levels than controls or to show no significant differences. Current PET studies in SUD are limited by TSPO nondisplaceable binding and competition from metabolites involved in essential mitochondrial functions (e.g., cholesterol transport). Future research with longitudinal designs or new promising PET radiotracers could elucidate the effects of SUD on neuroimmune signaling. For example, newer PET tracers that target COX-1 and COX-2 or inducible nitric oxide synthase (iNOS) were found to be sensitive to neuroinflammation in individuals with Alzheimer’s disease (110), Parkinson’s disease (111), and lung inflammation in electronic cigarette users (112). These tracers may be promising for detecting neuroinflammation in SUDs. Furthermore, MRI research on inflammation consistently showed increased levels of mI in brain, measured using ^1^H-MRS (e.g., meta-analyzed in 39), and lower FA in drug users compared with controls. However, these MRI measures do not directly assess neuroinflammation markers but rather downstream consequences (e.g., neuronal dysfunction, membrane turnover, and white matter microstructure). Future studies that combine PET and MRI modalities and associate them with peripheral markers of inflammation will help to clarify the role of neuroinflammation in modulating drug reward and the neuroadaptations resulting from acute drug administration, chronic drug exposure, and treatment for SUDs.

Several antiinflammatory medications have shown promise as treatment options for SUD. These include medications that target phosphodiesterases (PDEs), which comprise a large family of enzymes that regulate intracellular levels of secondary messengers, cAMPs, which are subsequently involved in the initiation and progression of inflammatory pathways. Inhibition of PDEs with the medications apremilast and ibudilast decreased markers of inflammation (113–115) as well as alcohol craving and consumption in preclinical and clinical studies (116–118). Preclinical studies also found that ibudilast treatment attenuated cocaine and methamphetamine self-administration in rodents (119, 120). Among non-treatment-seeking individuals with OUD, ibudilast attenuated subjective liking for oxycodone and reduced oxycodone self-administration (121). However, in treatment-seeking individuals with MUD, the effects on methamphetamine abstinence did not differ between those of ibudilast and placebo (122). Further elucidation of the clinical and neurobiological implications of treating SUD with ibudilast is warranted. Findings that apremilast decreases excessive drinking in individuals with AUD require replication (117). It should be noted that, in addition to modulating inflammatory pathways, PDE/cAMP signaling also regulates dopamine transmission (123), which may have contributed to ibudilast or apremilast’s effects on SUD. In a preclinical study, administration of PDE4 inhibitor rolipram mitigated cocaine-induced disruption in the balance between dopaminergic excitation/inhibition in the VTA and attenuated behavioral responses to cocaine (124). Therefore, future studies are warranted to better delineate the mechanisms underlying the actions of PDE inhibitors in SUD. The radioligand ^18^F-PF-06445974 was recently developed to quantify PDE isozyme 4B (PDE4B) (125), and it may be ideal for evaluating the effects of apremilast and ibudilast in individuals with SUD. Furthermore, N-acetyl cysteine (NAC), an antioxidant that has antiinflammatory properties and modulates glutaminergic signaling, is also being studied for the treatment of SUD (126). Preclinical studies have shown NAC to be effective in reducing drug-seeking behaviors (127), but clinical findings have been inconclusive. In a clinical trial conducted in individuals with AUD, NAC did not significantly differ from placebo in reducing alcohol consumption (128), whereas in a larger sample of individuals with cannabis use disorder, NAC administration was associated with increased odds of weekly alcohol abstinence and fewer drinking days in comparison to placebo (129). Future PET and MRI clinical treatment trials in individuals with SUDs that capture both the clinical and neuroimaging effects of antiinflammatory drug treatments are warranted.

## Figures and Tables

**Table 5:**
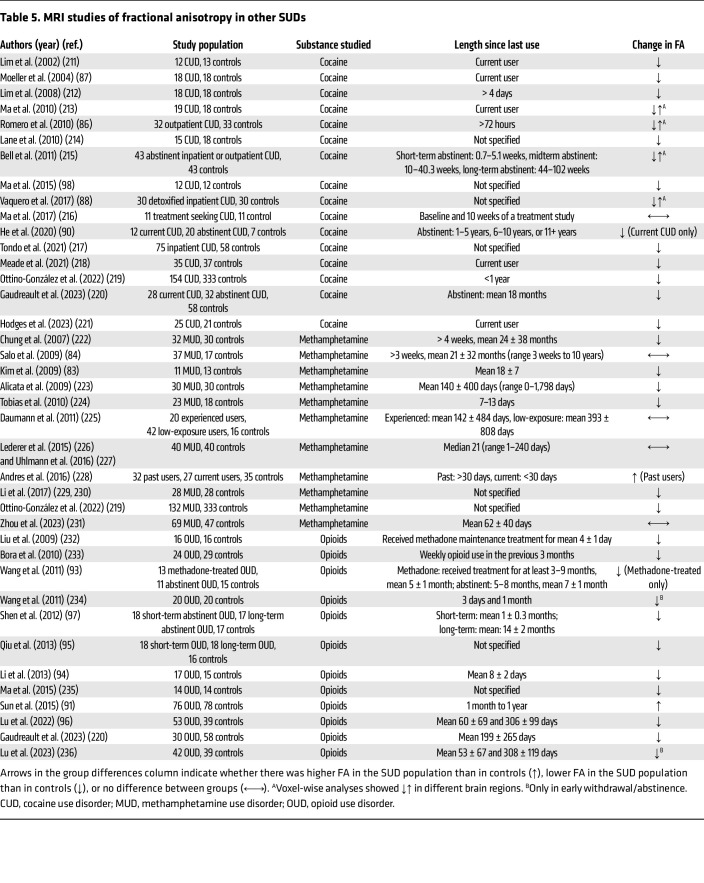
MRI studies of fractional anisotropy in other SUDs

**Table 4:**
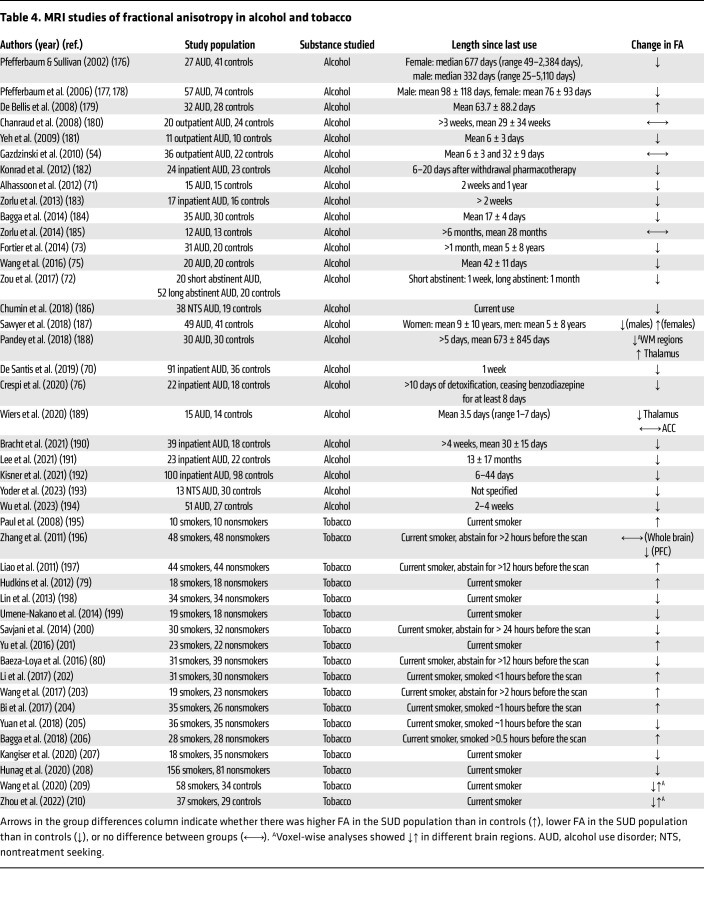
MRI studies of fractional anisotropy in alcohol and tobacco

**Table 3:**
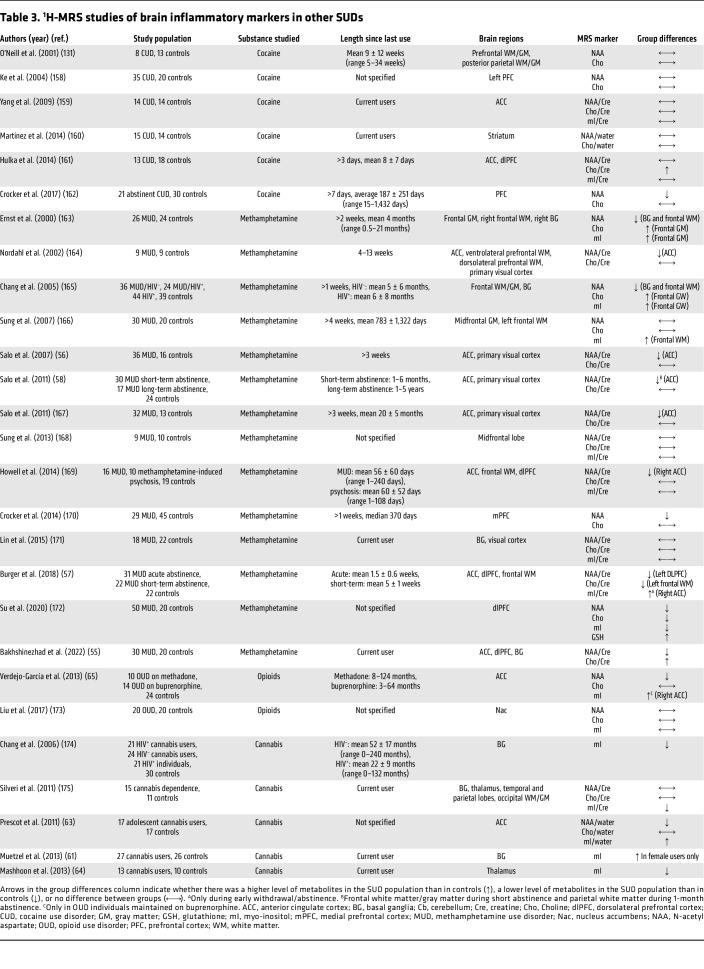
^1^H-MRS studies of brain inflammatory markers in other SUDs

**Table 2:**
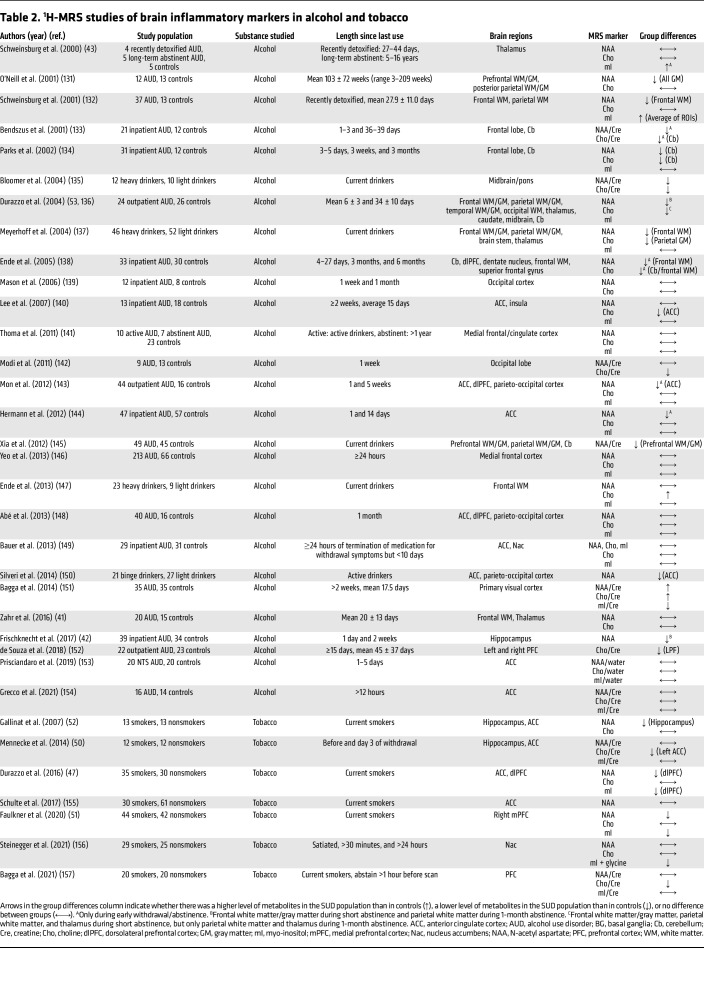
^1^H-MRS studies of brain inflammatory markers in alcohol and tobacco

**Table 1:**
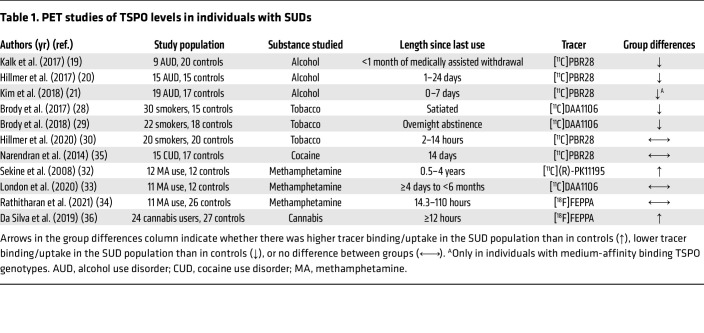
PET studies of TSPO levels in individuals with SUDs

## Version 1 06/03/2024

Electronic publication
